# Supplementary material for: Integrated Analysis of Tissue-Specific Gene Expression in Diabetes by Tensor Decomposition Can Identify Possible Associated Diseases
Source: Genes (Basel). 2022 Jun 20;13(6):1097. doi: 10.3390/genes13061097 (PMC9222230; doi:10.3390/genes13061097)
Supplement: Supplementary file 1 [file genes-13-01097-s001.zip › genes-1739228-supplementary/Supplementary_Information.pdf]

Supplementary Information

1 The brief description of the HOSVD algorithm

For demonstration, consider the tensor $x_{ijk} \in \mathbb{R}^{N \times M \times K}$. HOSVD is an algorithm that applies SVD to the unfolded tensor, either $x_{i(jk)} \in \mathbb{R}^{N \times MK}$, $x_{j(ik)} \in \mathbb{R}^{M \times NK}$, or $x_{k(ij)} \in \mathbb{R}^{K \times NM}$,

$$x_{i(jk)} = \sum_{\ell_1=1}^{\min(N,MK)} \lambda_{\ell_1} u_{\ell_1 i} v_{\ell_1 jk} \tag{1}$$

$$x_{j(ik)} = \sum_{\ell_2=1}^{\min(M,NK)} \lambda_{\ell_2} u_{\ell_2 j} v_{\ell_2 ik} \tag{2}$$

$$x_{k(ij)} = \sum_{\ell_3=1}^{\min(K,NM)} \lambda_{\ell_3} u_{\ell_3 k} v_{\ell_3 ij} \tag{3}$$

$$\tag{4}$$

where $u_{\ell_1 i} \in \mathbb{R}^{\min(N,MK) \times N}$, $v_{\ell_1 jk} \in \mathbb{R}^{\min(N,MK) \times MK}$, $u_{\ell_2 j} \in \mathbb{R}^{\min(M,NK) \times M}$, $v_{\ell_2 ik} \in \mathbb{R}^{\min(M,NK) \times NK}$, $u_{\ell_3 k} \in \mathbb{R}^{\min(K,MM) \times K}$, $v_{\ell_3 ij} \in \mathbb{R}^{\min(K,NM) \times NM}$. A core tensor $G(\ell_1 \ell_2 \ell_3)$ can be calculated as

$$G(\ell_1 \ell_2 \ell_3) = \sum_{i=1}^{N} \sum_{j=1}^{M} \sum_{k=1}^{K} x_{ijk} u_{\ell_1 i} u_{\ell_2 j} u_{\ell_3 k}. \tag{5}$$

Subsequently, we obtain the following

$$x_{ijk} = \sum_{\ell_1=}^{N} \sum_{\ell_2=1}^{M} \sum_{\ell_3=1}^{K} G(\ell_1 \ell_2 \ell_3) u_{\ell_1 i} u_{\ell_2 j} u_{\ell_3 k} \tag{6}$$

where $u_{\ell_1 i} \in \mathbb{R}^{N \times N}$, $u_{\ell_2 j} \in \mathbb{R}^{M \times M}$, and $u_{\ell_3 k} \in \mathbb{R}^{K \times K}$. When $u_{\ell_1 i}$, $u_{\ell_2 j}$, and $u_{\ell_3 k}$, are smaller than those computed in eqs. (1), (2), and (3), missing values are filled with zero. Then, $u_{\ell_1 i}$, $u_{\ell_2 j}$, and $u_{\ell_3 k}$ are the resultant orthogonal matrices obtained.

2 TD-based unsupervised FE with SD optimization

HOSVD was applied to x_{ijkmst}, and we obtained the following:

$$x_{ijkmst} = \sum_{\ell_1=1}^{5}\sum_{\ell_2=1}^{5}\sum_{\ell_3=1}^{2}\sum_{\ell_4=1}^{2}\sum_{\ell_5=1}^{3}\sum_{\ell_6=1}^{31099} G(\ell_1\ell_2\ell_3\ell_4\ell_5\ell_6) u_{\ell_1 j} u_{\ell_2 k} u_{\ell_3 m} u_{\ell_4 s} u_{\ell_5 t} u_{\ell_6 i} \tag{7}$$

where $G(\ell_1\ell_2\ell_3\ell_4\ell_5\ell_6) \in \mathbb{R}^{5\times5\times2\times2\times3\times31099}$ is a core tensor and $u_{\ell_1 j} \in \mathbb{R}^{5\times5}$, $u_{\ell_2 k} \in \mathbb{R}^{5\times5}$, $u_{\ell_3 m} \in \mathbb{R}^{2\times2}$, $u_{\ell_4 s} \in \mathbb{R}^{2\times2}$, $u_{\ell_5 t} \in \mathbb{R}^{3\times3}$, and $u_{\ell_6 i} \in \mathbb{R}^{31099\times31099}$ are singular value matrices and orthogonal matrices.

After identifying $u_{\ell_1 j}$, $u_{\ell_2 k}$, $u_{\ell_3 m}$, $u_{\ell_4 s}$, and $u_{\ell_5 t}$ of interest, we attempted to identify $G(\ell_1\ell_2\ell_3\ell_4\ell_5\ell_6)$ with the largest absolute value and fixed ℓ_1 to ℓ_5. Then, we attributed P-values to i by assuming that $u_{\ell_6 i}$ obeys a Gaussian distribution as follows:

$$P_i = P_{\chi^2}\left[> \left(\frac{u_{\ell_6 i}}{\sigma_{\ell_6}}\right)^2 \right] \tag{8}$$

where $P_{\chi^2}[> x]$ is the cumulative χ^2 distribution under the assumption that it is larger than x and σ_{ℓ_6} is the SD. P_i is corrected with the BH criterion [?].

The SD is optimized so that $u_{\ell_6 i}$ obeys the Gaussian distribution (null hypothesis) as much as possible [?], and the optimization process is as follows. Initially, we compute the histogram h_p, which includes the number of i values that satisfy the following:

$$\frac{p}{N_p} \leq 1 - \text{adjusted}P_i \leq \frac{p+1}{N_p}, 0 \leq p \leq N_p - 1 \tag{9}$$

Then, the standard deviation of h_p for adjusted$P_i > p_0$ for the threshold value p_0,

$$\Delta h_p = \frac{1}{N'_p} \sum_{\text{adjusted}\frac{p}{N_P} > p_0} (h_p - \langle h_p \rangle)^2 \tag{10}$$

$$\langle h_p \rangle = \frac{1}{N'_p} \sum_{\text{adjusted}\frac{p}{N_P} > p_0} h_p \tag{11}$$

$$N'_P = \sum_{\text{adjusted}\frac{p}{N_P} > p_0} 1 \tag{12}$$

is minimized. Therefore, for $1 - \text{adjusted}P_i < 1 - p_0$, h_p takes a constant value (i.e., it obeys the null hypothesis) as much as possible, whereas for $1 - \text{adjusted}P_i > 1 - p_0$, h_p presents a peak value.

The P_i values are finally calculated using the optimized SD, and i values associated with adjusted P-values less than 0.01 are selected. Probes, is, are converted to gene symbols using the gene ID conversion tool in DAVID [?].

The genes selected are presumed to be coincident with the profiles associated with selected $u_{\ell_1 j}$, $u_{\ell_2 k}$, $u_{\ell_3 m}$, $u_{\ell_4 s}$, and $u_{\ell_5 t}$; this corresponds to the identification of differentially expressed genes.

For details, check sample R source code in supplementary materials.

3 Gene sepections

The results indicated that u_{2j} represents the dependence on time, u_{2m} represents the distinction between the controls and treatment, u_{1k} represents the independence of the replicate, u_{1s} represents the independence of strains, and u_{1t} represents the independence of tissues (Fig. S1). Table S1 presents

Table S1: The core tensor defined in eq. (7) when HOSVD is applied to data sets, $G(2,1,2,1,1,\ell_6)$.

ℓ_6	$G(2,1,2,1,1,\ell_6)$	ℓ_6	$G(2,1,2,1,1,\ell_6)$
1	3.505367	6	6.227675
2	8.092320	7	6.890919
3	-15.707937	8	-3.287872
4	9.134575	9	-1.529140
5	6.723113	10	-9.158979

$G(2,1,2,1,1,\ell_6)$, and it shows that $G(2,1,2,1,1,3)$ has the highest absolute value. Then, u_{3i} (i.e., $\ell_6 = 3$) is used to select probes. The P_i values are determined using eq. (8), with $\ell_6 = 3$. SD optimization was performed, and $\sigma_3 = 0.001514603$. A histogram of 1-P_i after SD optimization is presented in Figure S2 and the histogram is more similar to that of the null hypothesis, i.e., the combination of a flat region and a sharp peak. Then, the P-values corresponding to the ith probe are determined with eq. (8), and the P_i values are corrected by BH criterion. Finally, 2,452 selected probes are associated with adjusted P-values less than 0.01, and they are further converted to 2,281 gene symbols.

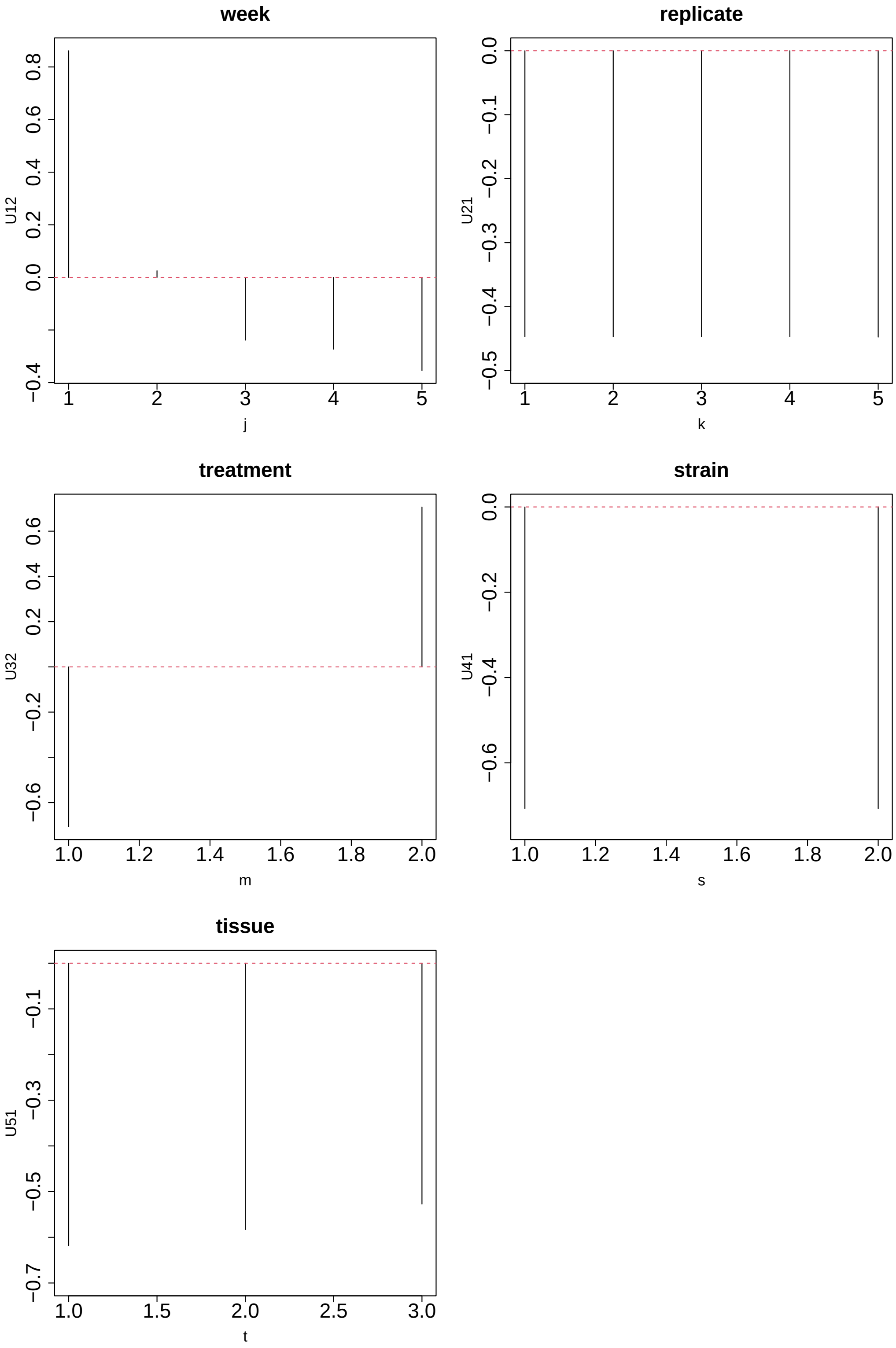

Figure S1: Singular value vectors defined in eq. (7) when HOSVD is applied to data sets. u_{2j} (week), u_{1k} (replicate), u_{2m} (treatment), u_{1s} (strain), and u_{1t} (tissue).

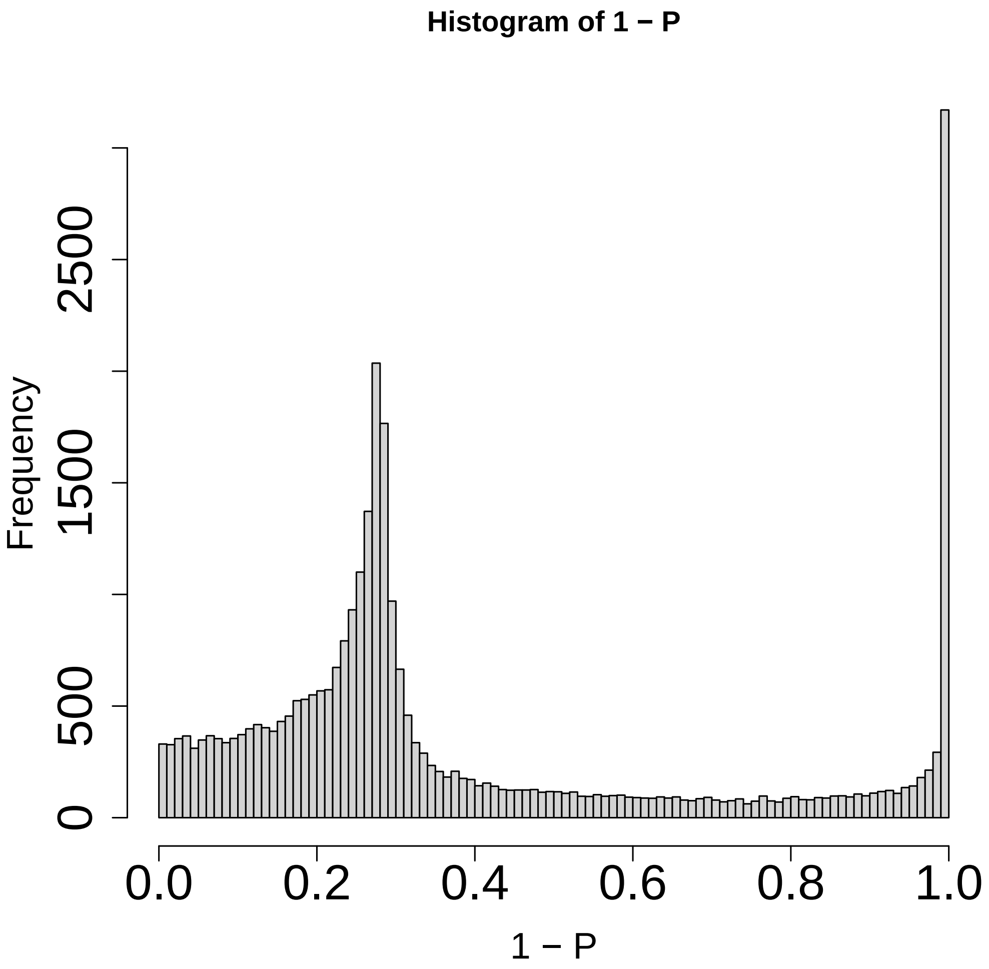

Figure S2: Histogram of 1-P_i.
